# Deletion of tumor suppressors adenomatous polyposis coli and Smad4 in murine luminal epithelial cells causes invasive prostate cancer and loss of androgen receptor expression

**DOI:** 10.18632/oncotarget.17919

**Published:** 2017-05-17

**Authors:** Kenneth C. Valkenburg, Angelo M. De Marzo, Bart O. Williams

**Affiliations:** ^1^ Center for Cancer and Cell Biology, Van Andel Research Institute, Grand Rapids, MI 49503, USA; ^2^ Department of Pathology, Johns Hopkins School of Medicine, Baltimore, MD 21287, USA; ^3^ Department of Oncology, Johns Hopkins School of Medicine, Baltimore, MD 21287, USA

**Keywords:** mouse model, prostate cancer, basaloid, squamous, Apc

## Abstract

Prostate cancer is the most diagnosed non-skin cancer in the US and kills approximately 27,000 men per year in the US. Additional genetic mouse models are needed that recapitulate the heterogeneous nature of human prostate cancer. The Wnt/beta-catenin signaling pathway is important for human prostate tumorigenesis and metastasis, and also drives tumorigenesis in mouse models. Loss of Smad4 has also been found in human prostate cancer and drives tumorigenesis and metastasis when coupled with other genetic aberrations in mouse models. In this work, we concurrently deleted Smad4 and the tumor suppressor and endogenous Wnt/beta-catenin inhibitor adenomatous polyposis coli (Apc) in luminal prostate cells in mice. This double conditional knockout model produced invasive castration-resistant prostate carcinoma with no evidence of metastasis. We observed mixed differentiation phenotypes, including basaloid and squamous differentiation. Interestingly, tumor cells in this model commonly lose androgen receptor expression. In addition, tumors disappear in these mice during androgen cycling (castration followed by testosterone reintroduction). These mice model non-metastatic castration resistant prostate cancer and should provide novel information for tumors that have genetic aberrations in the Wnt pathway or Smad4.

## INTRODUCTION

Prostate cancer is the most diagnosed non-skin cancer in men in the United States. Approximately 27,000 men die each year from prostate cancer [1], yet many men survive for years with indolent forms of the disease. Distinguishing indolent from aggressive prostate cancer is a high priority. As such, a need exists for experimental models that recapitulate progression of human prostate cancer. Many molecular signaling pathways are altered in human prostate cancer, meaning that multiple genetic models must exist to ascertain the tumorigenic contributions of these pathways and to generate targeted therapies against them.

Wnt/β-catenin pathway activation correlates with prostate cancer progression and metastasis [2]. Notably, a multi-institutional effort to sequence metastatic, castration-resistant prostate cancer found that genomic alterations in the Wnt signaling pathway occur in approximately 18% of cases, and these included alterations in adenomatous polyposis coli (Apc), an endogenous inhibitor of Wnt signaling [3]. Conditional activation of the Wnt/β-catenin pathway in the mouse, either via knockout of Apc [4, 5] or constitutive activation of β-catenin [6, 7], causes high grade prostate intraepithelial neoplasia (HGPIN) with infrequent invasiveness. These data indicate that Wnt/β-catenin signaling activation alone is sufficient for the development of pre-invasive lesions, but insufficient for progression to invasive or metastatic cancer. A second genetic hit is likely necessary to induce progression [8].

Smad4 is known as the common Smad or Co-Smad because of its necessity in both bone morphogenetic protein (BMP) and transforming growth factor beta (TGFβ) signaling. Smad4 is a tumor suppressor in prostate tissue [9–11]. Smad4 is deleted in approximately 10% of prostate cancer cases. It is co-deleted with tumor suppressors phosphatase and tensin homolog (Pten) and p53 approximately 55% of the time it is mutated [10]. Smad4 loss promotes progression and metastasis in murine prostate cancer models, including conditional Pten and p53 knockouts [10, 12].

The Wnt pathway and Smad4 have been linked in prostate cancer. Oncogenic microRNA miR-1260b targets both Smad4 and secreted frizzled-related protein 1 (sFRP1, an endogenous Wnt pathway inhibitor) [13], suggesting that there is a population of prostate cancer patients in which the Wnt pathway is up-regulated while the BMP and TGFβ pathways are inactivated. Another study showed that Wnt3a-neutralizing antibodies reversed tumorigenesis caused by the stromal-specific knockout of TGFβ receptor II, which is frequently lost in human prostate cancer [14].

Because of these data, we created a genetically engineered mouse model in which Apc and Smad4 were concurrently deleted in prostate luminal cells, using the *Nkx3.1* gene promoter to drive tamoxifen-inducible Cre recombinase expression [15]. We and others have shown that *Nkx3.1*^CreERT^ is active in the luminal cell population of the adult murine prostate [5, 15]. We show here that loss of both Apc and Smad4 results in invasive, castration-resistant prostate carcinoma with androgen receptor (AR) loss. Notably, this phenotype disappeared upon hormonal regeneration following castration.

## RESULTS

### Apc – Smad4 double knockout mouse model

**Figure 1:**
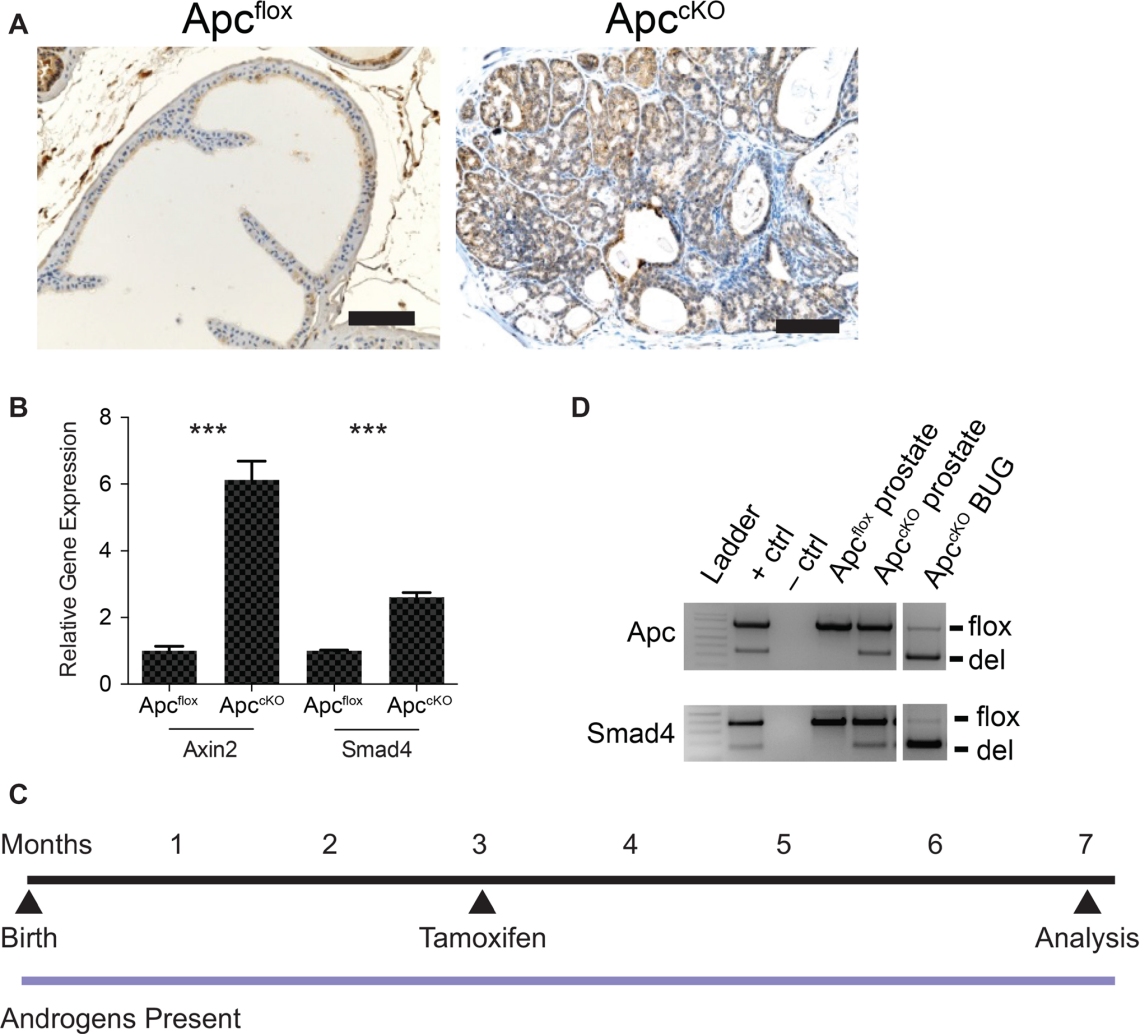
Rationale behind the Apc-Smad4 double knockout (Apc^cKO^Smad4^cKO^) mouse model (**A**) Smad4 immuno-staining on prostate tissue from Apc^flox^ and Apc^cKO^ mice (scale bars = 100 μm); (**B**) *Axin2* and *Smad4* quantitative reverse-transcriptase PCR from Apc^flox^ and Apc^cKO^ prostate tissue (error bars represent standard deviation based on technical triplicates; ***P value < 1 × 10^−4^); (**D**) allele-specific PCR for Apc and Smad4 showing the floxed band (flox) and the band as a result of deletion (del) in prostate and bulbourethral gland tissue; and (**C**) timeline of tamoxifen administration and sacrifice of control and experimental mice.

Smad4 protein levels in wild-type prostate tissue are relatively low, but when the tumor suppressor phosphatase and tensin homolog (Pten) is lost, Smad4 levels increase, suggesting that Smad4 acts to constrain tumor progression [10]. To determine whether Smad4 levels would also increase in a Wnt/ β-catenin-driven tumor model using the *Nkx3.1*^CreERT^ model (*Nkx3.1*^CreERT^*Apc*^flox^, referred to as Apc^cKO^ [5]), we performed Smad4 immunohistochemistry (IHC) and quantitative reverse transcriptase PCR (qPCR) on Cre-negative (Apc^flox^) and Cre-positive (Apc^cKO^) prostate tissue. Smad4 protein and RNA levels increased in Apc^cKO^ mice relative to the Apc^flox^ control (Figure 1A–1B). Axin2 expression also increases upon Apc loss as expected, and served as a positive control (Figure 1B). We concluded from these data that Smad4 might be constraining tumor progression in Apc^cKO^ mice, which display high-grade prostate intraepithelial neoplasia (HGPIN) but infrequent invasion and no evidence of metastasis [5]. Therefore, we crossed Apc^cKO^ mice to Smad4^flox^ mice, a generous gift from Ronald DePinho (MD Anderson Cancer Center) [16]. We will refer to these *Nkx3.1*^CreERT^*Apc*^flox^*Smad4*^flox^ double knockout mice as Apc^cKO^Smad4^cKO^, in which both the *Apc* and *Smad4* genes have been deleted in the prostate. It is important to note that the Cre driver we used (*Nkx3.1*^CreERT^) is tamoxifen-inducible, meaning that Cre recombinase is not active until mice are treated with tamoxifen; we treated 3-month-old mice once per day for two consecutive days via oral gavage to activate Cre (Figure 1C) [5]. We used Cre-negative mice bearing the Apc and Smad4 floxed alleles as controls, and we will refer to them as Apc^flox^Smad4^flox^ mice. We also treated these control mice with tamoxifen; the only thing lacking in the control mice was Cre recombinase. We have previously shown that treating *Nkx3.1*^CreERT^*Apc*^wild-type^ mice with tamoxifen showed no overt phenotype [5]. Using allele-specific PCR we determined that in addition to Apc recombination [5], we were able to induce Smad4 recombination in prostate tissue (Figure 1D). Genetic recombination did not occur in lung, liver, lymph node, or testis (data not shown), but did occur in the bulbourethral gland (Figure 1D). This is due to Nkx3.1 expression in the bulbourethral gland (BUG) as previously reported [4, 17].

### Invasive carcinoma in *Apc^cKO^Smad4^cKO^* mice

**Figure 2:**
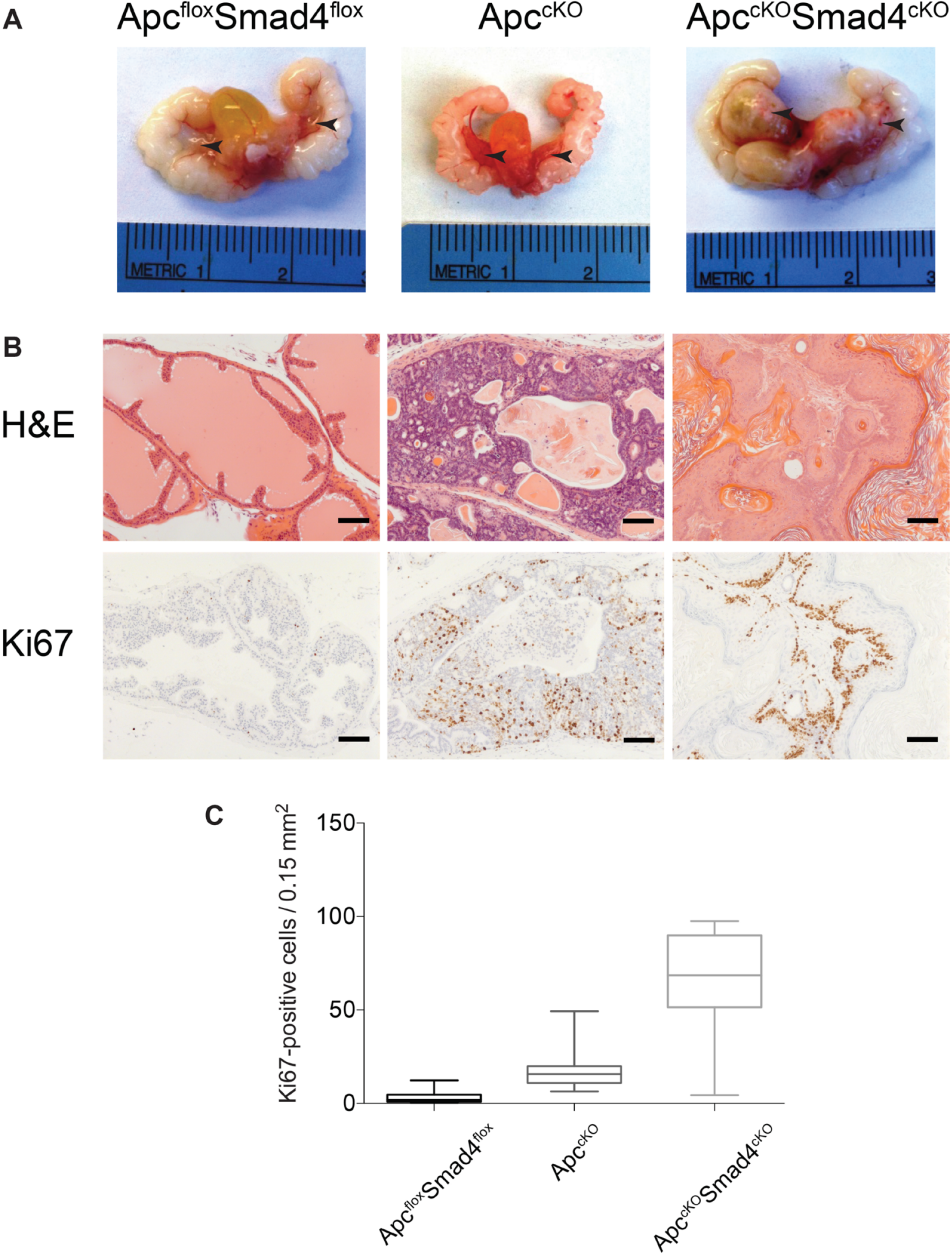
Proliferative increases in Apc^cKO^Smad4^cKO^ mice compared to Apc^cKO^ mice (**A**) Urogenital tracts, including prostate tissue, from Apc^flox^Smad4^flox^, Apc^cKO^, and Apc^cKO^Smad4^cKO^ mice (arrows indicate the anterior prostate lobes); (**B**) H&E staining and Ki67 immuno-staining of prostate tissues (scale bars = 100 μm); and (**C**) Nuance microscope quantification of proliferating (Ki67-positive) cells in prostate cells from Apc^flox^Smad4^flox^, Apc^cKO^, and Apc^cKO^Smad4^cKO^ mice. For all groups, *P* value < 1.0 ×10^−12^.

We euthanized Apc^cKO^Smad4^cKO^ mice at 7 months of age and observed large tumors in the anterior prostate lobes in 72% (13/18) of the mice (Figure 2A). It is possible that some of the mice did not get tumors due to a mixed genetic background (Supplementary Table 1). No overt tumors were grossly apparent in Apc^cKO^ or Smad4^cKO^ prostates, as previously observed (although Apc^cKO^ mice did develop high-grade prostate intraepithelial neoplasia (HGPIN)) [5, 10]. Smad4^cKO^ prostate tissue displayed no histological evidence of neoplasia (Supplementary Figure 3). Apc^cKO^Smad4^cKO^ mice did not typically survive past 10 months of age due to large tumors in their abdomen, which originated from the BUG (Supplementary Figure 2A). Apc^cKO^Smad4^cKO^ tumors were extremely proliferative, as evidenced by a statistically significant rise in Ki67 staining relative to Apc^flox^Smad4^flox^ control tissue or Apc^cKO^ tumors (Figure 2B–2C).

**Figure 3:**
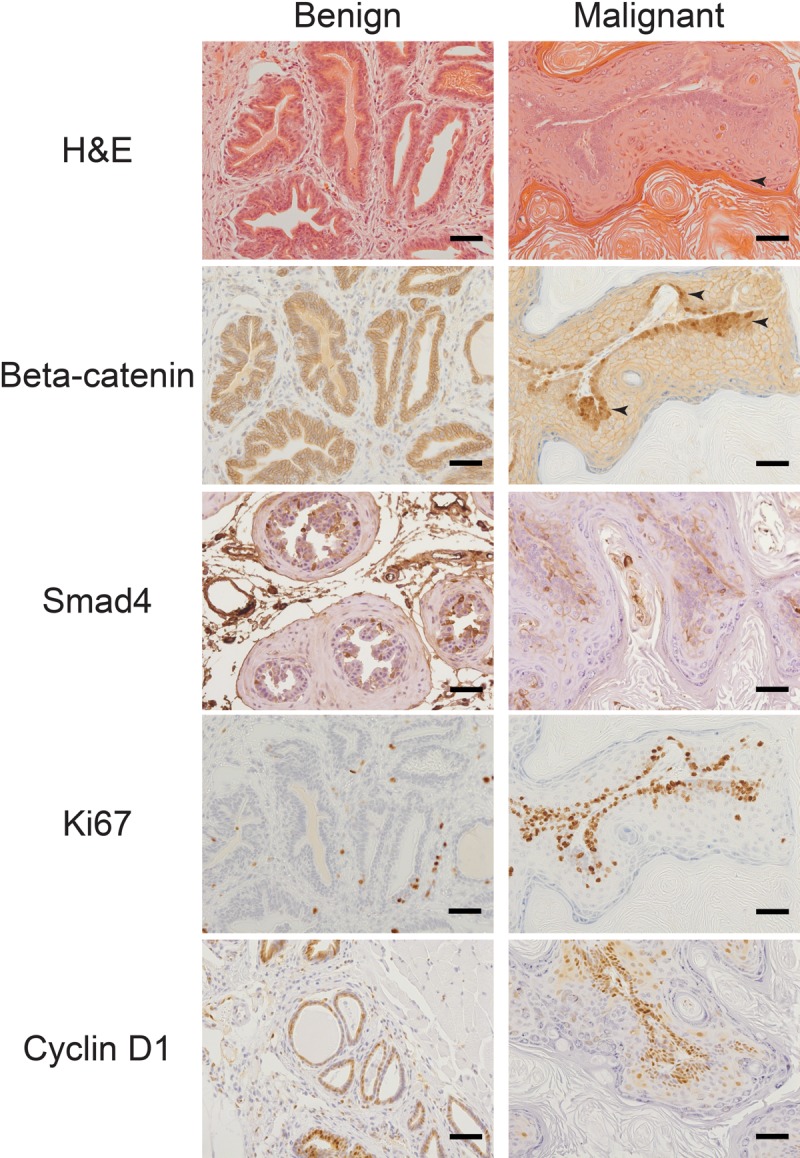
Gain of beta-catenin expression plus loss of Smad4 expression is associated with increased proliferation Benign and malignant prostate tissue from Apc^cKO^Smad4^cKO^ mice were stained for hematoxylin and eosin (H&E) and immuno-stained for beta-catenin, Smad4, Ki67, and cyclin D1. Arrows point to regions keratohyalin granule formation and loss of nuclei (H&E stain) and regions of nuclear beta-catenin staining. Scale bars = 50 μm.

**Figure 4:**
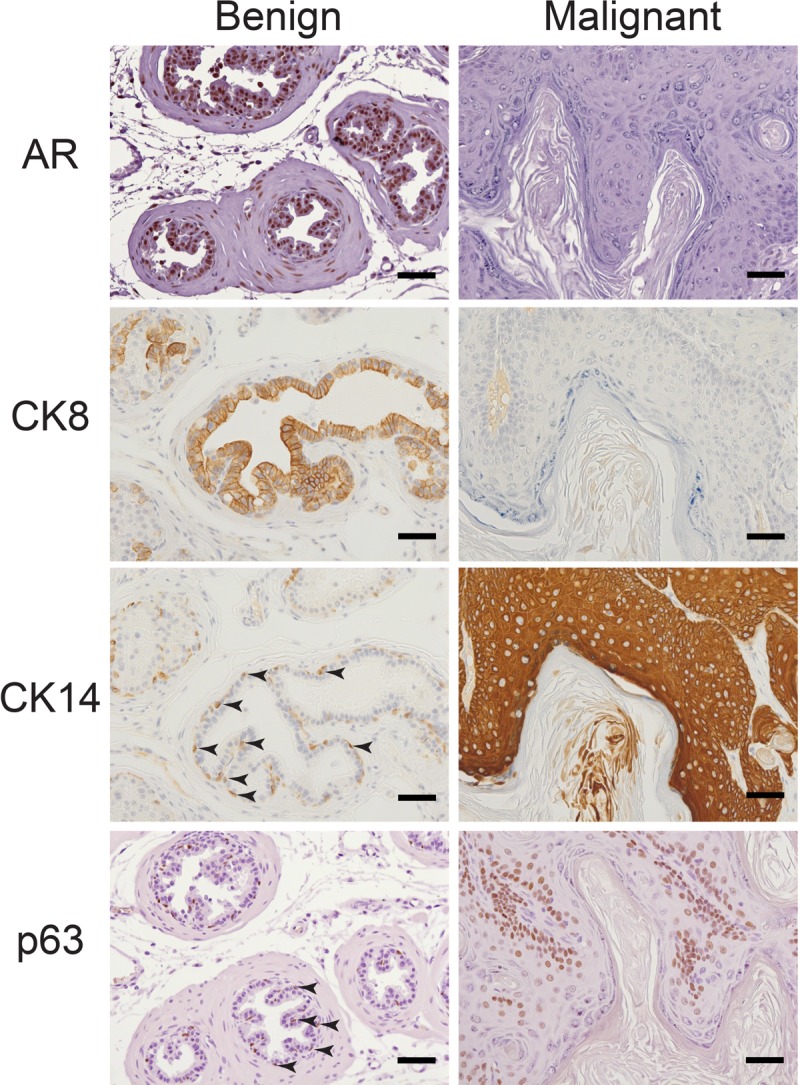
Concurrent loss of AR and luminal marker CK8 and expansion of basal markers CK14 and p63 in malignant tissue Benign and malignant prostate tissue from Apc^cKO^Smad4^cKO^ mice were immuno-stained for androgen receptor (AR), cytokeratin 8 (CK8), cytokeratin 14 (CK14), and p63. Arrows point to representative positively stained CK14 and p63 basal cells. Scale bars = 50 μm.

Further histological analysis revealed that Apc^cKO^Smad4^cKO^ tumors displayed a range of differentiation phenotypes. We observed large regions of squamous differentiation with keratohyalin granule formation and loss of nuclei (Figure 3). As expected, cytoplasmic and nuclear β-catenin levels remained low in Apc^flox^Smad4^flox^ prostate tissue but rose in the basal cell compartment of Apc^cKO^Smad4^cKO^ malignant prostate tissue (Figure 3). The gain of nuclear and cytoplasmic β-catenin and loss of Smad4 directly correlated to increased levels of proliferation markers Ki67 and cyclin D1 (Figure 3). The bulbourethral glands from Apc^cKO^Smad4^cKO^ mice also displayed invasive carcinoma, increased β-catenin signaling, and high levels of proliferation (Supplementary Figure 2B). Interestingly, we observed a loss of AR expression in malignant prostate tissue (Figure 4). Changes in AR expression have not been linked to loss of Apc or Smad4 previously. We also assessed expression of luminal cell marker cytokeratin 8 (CK8) and basal cell markers cytokeratin 14 (CK14) and p63. Malignant prostate tissue lost CK8 expression and gained CK14 and p63 expression (Figure 4). p63 expression was located largely in the basal cell layer in Apc^flox^ Smad4^flox^ mice, but expanded significantly in Apc^cKO^Smad4^cKO^ mice (Figure 4).

**Figure 5:**
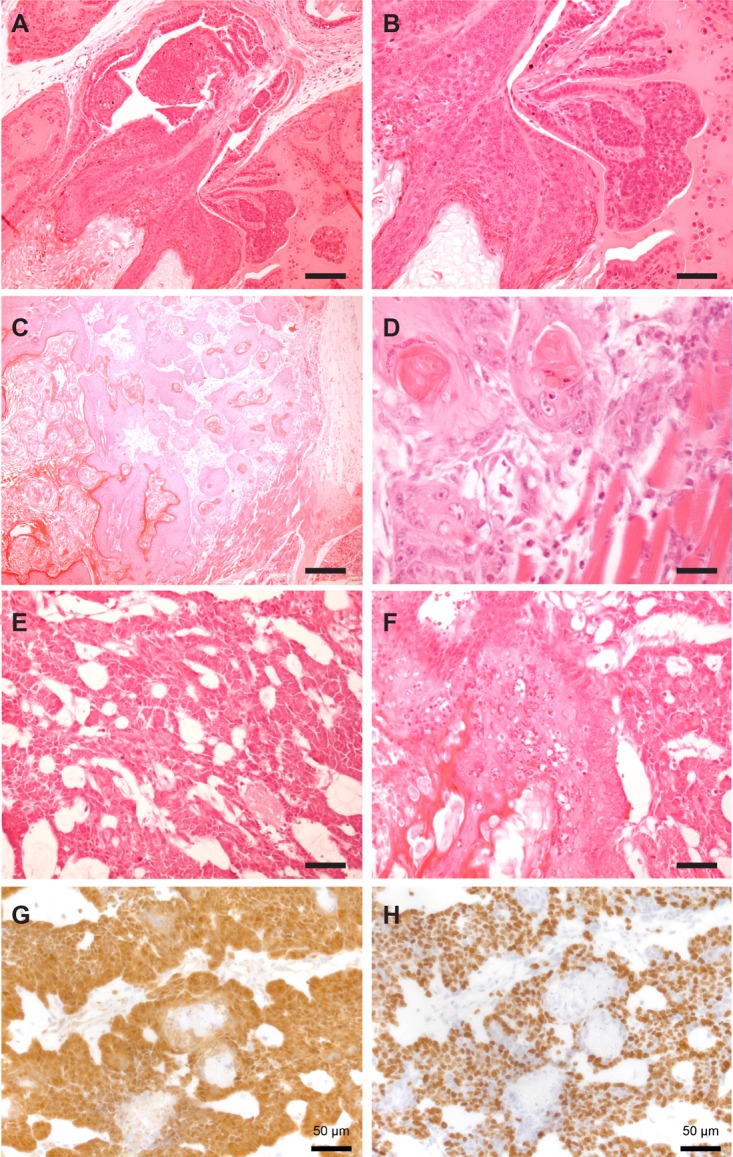
Mix of differentiation phenotypes in Apc^cKO^Smad4^cKO^ tumors Representative hematoxylin and eosin staining of prostate tumors from Apc^cKO^Smad4^cKO^ mice. Squamous *in situ* carcinoma (**A**–**B**); scale bars = 100 μm (A) and 200 μm (B). Squamous carcinoma with muscular invasion (**C**–**D**); scale bars = 250 μm (C) and 25 μm (D). Basaloid carcinoma (**E**); scale bar = 50 μm. Representative image of mixed basaloid and squamous cell carcinoma in the same region (**F**); scale bar = 50 μm. Representative images of basaloid region of prostate tumor stained with beta-catenin (**G**) and Ki67 (**H**); scale bars = 50 μm.

In addition to squamous differentiation, we also observed carcinoma with a basaloid appearance and cribriform architecture (Figure 5). We observed invasion of both basaloid and squamous carcinomas into the muscle layer (Figure 5C–5D). Figure 5F shows a region of both differentiation phenotypes in the same small region of a tumor. While β-catenin and Ki67 staining was localized to the basal layer in squamous regions (Figure 3), basaloid regions had pervasive β-catenin and Ki67 staining patterns, more similar to what was seen in the Apc^cKO^ model (Figure 5G–5H).

Interestingly, Smad4^cKO^ prostate tissue displayed normal features when stained for β-catenin, Ki67, CK8, CK14, cyclin D1, and AR (Supplementary Figure 3), indicating that loss of both tumor suppressors is required to cause this observed phenotype. These data indicate that deletion of Apc and Smad4 are indeed the driving forces behind the carcinomatoid phenotype observed in these mice.

### Tumors in *Apc^cKO^Smad4^cKO^* mice are castration resistant

**Figure 6:**
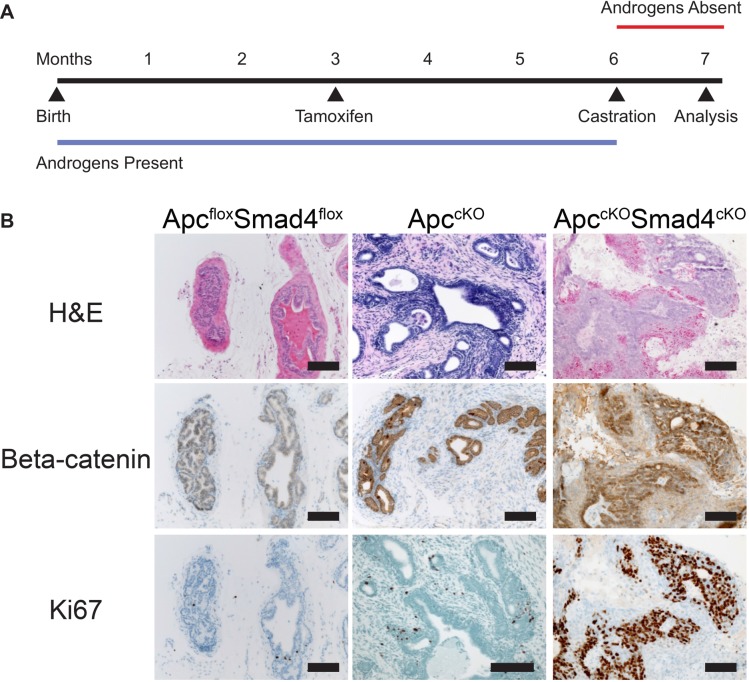
Tumors in Apc^cKO^Smad4^cKO^ mice are castration resistant (**A**) Timeline of tamoxifen administration, castration, and sacrifice of control and experimental mice; and (**B**) representative images of prostate tissue from Apc^flox^Smad4^flox^, Apc^cKO^, and Apc^cKO^Smad4^cKO^ mice were stained for hematoxylin and eosin (H&E) and immuno-stained for beta-catenin and Ki67. Scale bars = 100 μm.

To test the ability of Apc^cKO^Smad4^cKO^ prostate tumors to grow under castration conditions, we treated mice with tamoxifen at 3 months of age, and then castrated them 3 months later, after tumors had presumptively formed (Figure 6A). Tumors shrunk moderately after castration but retained regions of nuclear β-catenin and Ki67 staining, indicating castration resistance (Figure 6B). The same phenotype was present in the BUGs in Apc^cKO^Smad4^cKO^ mice (Supplementary Figure 2C). When we castrated before tamoxifen administration, tumors did not form in the prostate or BUG, indicating that androgens are required for tumor development but not for tumor maintenance (data not shown). We had previously observed a similar phenotype in the Apc^cKO^ mice [4].

### Androgen cycling eliminates the carcinoma phenotype

**Figure 7:**
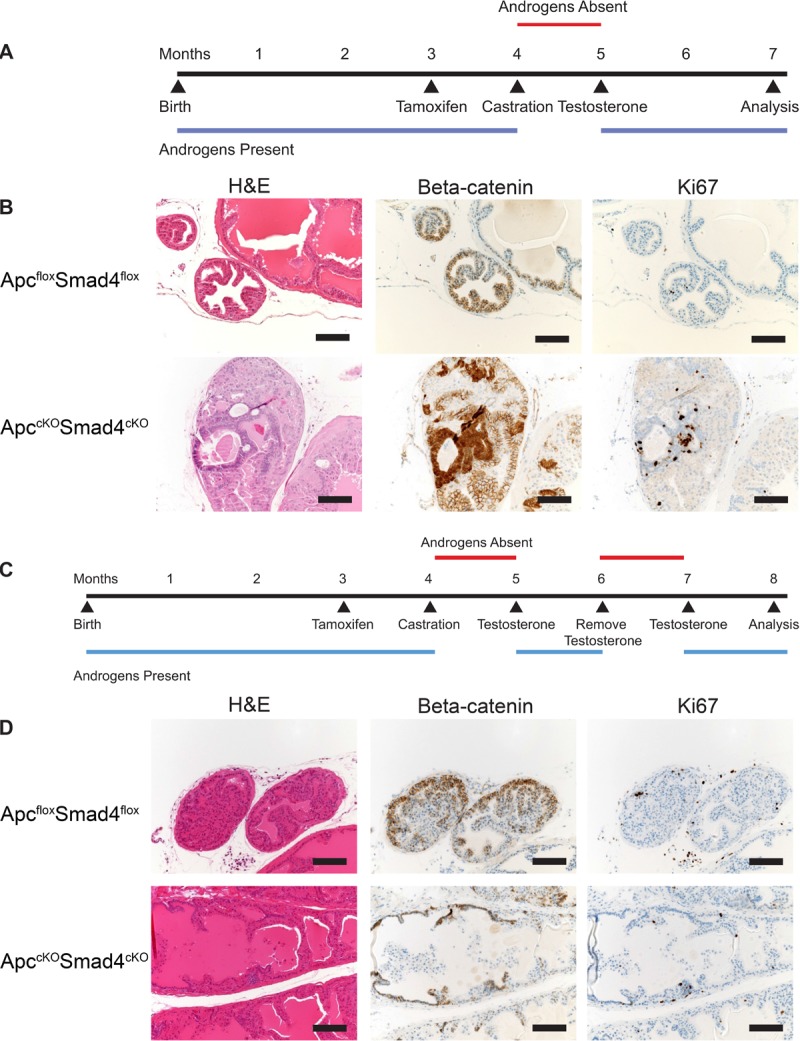
Loss of cancer phenotype after one or more rounds of androgen cycling Timelines of one rounds of androgen cycling (**A**) and two rounds of androgen cycling (**C**) in control and experimental mice. Representative hematoxylin and eosin (H&E), beta-catenin, and Ki67 staining of prostate tissue from Apc^flox^Smad4^flox^ and Apc^cKO^Smad4^cKO^ mice (**B** and **D**). Scale bars = 100 μm.

Wang et al. showed that a cycle of castration and testosterone reintroduction expanded the castration-resistant Nkx3.1-expressing cell (CARN) population, which behaves as a luminal-like stem cell [15]. We hypothesized that deleting Apc and Smad4 in this expanded stem-like population would cause aggressive carcinoma. However, we observed the opposite phenotype. Upon one cycle of castration and reintroduction of testosterone in Apc^cKO^Smad4^cKO^ mice (Figure 7A), we saw eradication of the invasive phenotype, replaced with occasional evidence of high-grade prostate intraepithelial neoplasia (HGPIN) (Figure 7B). After two cycles (Figure 7B), there was no longer any evidence of neoplasia in any of the prostate tissue. In addition, β-catenin expression remained membranous, indicating inactive Wnt signaling, and Ki67 staining indicated normal proliferation levels (Figure 7D). This phenotype was observed in the BUGs as well (Supplementary Figure 2D). We performed IHC for AR on prostate tissue from the various hormone states of the mice (hormonally normal, castrated, and androgen cycled). As reported earlier in this work, AR is lost in hormonally normal, tamoxifen treated Apc^cKO^Smad4^cKO^ prostate tissue (Supplementary Figure 1A). In castration resistant prostate tissue, we found strong AR staining in benign regions and loss of AR staining in malignant regions, suggesting that loss of AR is closely associated with malignancy in this model (Supplementary Figure 1B). Interestingly, AR staining appeared relatively normal in the androgen cycled Apc^cKO^Smad4^cKO^ prostate tissue (Supplementary Figure 1C). In addition, when we previously stained for AR in Apc^cKO^ lesions, we saw not only presence of AR, but an increase in AR staining [5]. This indicates that the combinatorial loss of both Apc and Smad4 produces the loss of AR phenotype.

## DISCUSSION

Multiple genetic pathways are involved in the tumorigenesis of the prostate [3, 18–21]. Due to this heterogeneity – as well as the normal physiological differences between human and mouse prostates [22, 23] – it has been difficult to create a mouse model that fully recapitulates human prostate cancer progression. Many genetically engineered models have been made, each one representing some aspect of prostate cancer, and contributing to the library of knowledge on the effect of each gene(s) on prostate tumorigenesis and progression [22]. Using similar strategies, we have generated a novel mouse model of invasive prostate carcinoma, in which Apc and Smad4 have been deleted in a specific population of luminal cells.

In previous work, we have shown that Apc deletion in luminal prostate cells, using one of two different prostate-specific promoters to drive Cre expression (Probasin or Nkx3.1), is tumorigenic, but is not sufficient to cause invasive carcinoma or metastasis [4, 5]. Others have shown similar results by activating a constitutive version of β-catenin using the same Cre drivers [6, 7]. These data suggest that at least a second genetic hit is required to induce invasiveness or metastasis. This appears counterintuitive, as Wnt/β-catenin activation in humans correlates with prostate cancer progression [2], though it is possible that other genetic hits are coincident in patients with up-regulated Wnt signaling.

We show for the first time within the prostate that loss of Apc and Smad4 act in concert to promote tumorigenesis and invasion. Apc loss by itself results in HGPIN, and Smad4 loss by itself results in no observable histological change. But when combined, large tumors form with extremely high levels of proliferation as well as invasive properties. Histological analysis of these tumors revealed a range of differentiation, including basaloid and squamous differentiation, rare phenotypes in human prostate cancer patients. It has been suggested that Apc and Smad4 may act in concert in humans, and two separate microRNAs have been found to target Smad4 in addition to Wnt signaling antagonists secreted frizzled related protein 1 (sFRP1) or Dickkopf 3 (DKK3) [13, 24]. They showed that DKK3 reduced TOP-Flash luciferase activity, indicating that, at least in this context, DKK3 reduced Wnt activity. This suggests that the Apc^cKO^Smad4^cKO^ mouse model may have value in determining the mechanisms behind the rare sub-types (basaloid and squamous) of prostate cancer.

Similar to our previously published Apc deletion models [4, 5], we observed castration resistance of the primary tumors of Apc^cKO^Smad4^cKO^ mice if mice were castrated after tumor development. If mice were castrated prior to tumor development (in our model, prior to tamoxifen administration) tumors did not develop. These data indicate that Apc^cKO^Smad4^cKO^ tumors rely on androgens for tumorigenesis, but once tumors develop, they grow in the absence of androgens. Wnt/β-catenin signaling has also been linked to AR signaling, and may serve to promote tumorigenesis in the absence of AR signaling, supporting this theory [25–27].

One unexpected phenomenon we observed in the Apc^cKO^Smad4^cKO^ model was the disappearance of tumors with androgen cycling (castration followed by re-introduction of testosterone). Androgen deprivation therapy (ADT) is a common treatment for metastatic prostate cancer, due to the dependence of the prostate and most prostate cancers on androgens. When patients develop castration resistant disease, their cancer is aggressive and incurable. One strategy that is currently in clinical trials is to administer supraphysiological amounts of testosterone following castration, in a similar hormone cycling manner that we employed. This strategy is known as rapid androgen cycling or bipolar androgen therapy [28–31].

While hormone depletion results in a high rate of apoptosis in prostate tumors, castration resistance eventually develops in response [28]. AR expression is elevated in response to castration via multiple mechanisms, including *AR* gene amplification and mutation [32–35]. Exposure of AR-positive prostate cancer cells to supraphysiological concentrations of androgen results in growth inhibition [36, 37]. A high concentration of androgen leads to transient double-stranded DNA breaks via recruitment of topoisomerase IIβ [38]. It has also been shown that AR plays a suppressive role in basal cell populations in the prostate, indicating that loss of AR may drive proliferation of a more basaloid phenotype [39]. Putting these ideas together, ADT plus supraphysiological androgen cycling seems to result in a delayed castration resistance, growth inhibition, and apoptosis due to DNA damage. While we observed a loss of AR in the malignant regions of the prostates (particularly the squamous regions) from Apc^cKO^Smad4^cKO^ mice, we observed that there were still benign, AR-expressing regions remaining (Supplementary Figure 1B). In addition, we have previously shown an increase of AR expression in response to Apc depletion alone [5]. We hypothesize that the combination of up-regulation of AR expression early in the model with hormone cycling resulted in loss of the tumor phenotype in our model.

The Apc^cKO^Smad4^cKO^ model will provide new genetic information for prostate cancer development, particularly in the basaloid and squamous differentiated cancers, and will serve as a model of castration resistance and potentially for a sub-population of prostate cancer patients whose tumors involve some combination of aberrant Wnt, TGFβ, and BMP signaling.

## MATERIALS AND METHODS

### Generation of mice

All animals were used in protocols that were reviewed and approved by the Institutional Animal Care and Use Committee of the Van Andel Research Institute. *Apc*^flox^ [4], *Smad4*^flox^ [16], and *Nkx3.1*^CreERT^ [15], have all been previously described. To generate Apc^cKO^ mice, we crossed *Nkx3.1*^CreERT^ heterozygous mice (transgenic allele plus wild-type allele, or tg/+) with mice homozygous for a floxed allele of *Apc* (flox/flox) to generate *Nkx3.1*^CreERTtg/+^*;Apc*^flox/+^ mice [5]. We then crossed these mice together to generate homozygous mutant *Nkx3.1*^CreERTtg/+^*;Apc*^flox/flox^ mice. Mice wild-type for Cre (*Nkx3.1*^CreERT+/+^*;Apc*^flox/flox^) were subjected to the same experimental conditions as the mutant mice and were used as controls. To generate Apc^cKO^Smad4^cKO^ mice, we crossed *Nkx3.1*^CreERTtg/+^*;Apc*^flox/flox^ mice to *Smad4*^flox/flox^ mice to generate *Nkx3.1*^CreERTtg/+^*;Apc*^flox/+^*;Smad4*^flox/+^ mice. We then crossed these mice together to generate homozygous mutant *Nkx3.1*^CreERTtg/+^*;Apc*^flox/flox^*:Smad4*^flox/flox^ mice. Cre-negative mice that were homozygous for Apc and Smad4 floxed alleles (*Nkx3.1*^CreERT+/+^*;Apc*^flox/flox^*:Smad4*^flox/flox^ which we refer to as Apc^flox^Smad4^flox^ mice) were used as negative controls. For genotyping of all mice, DNA was prepared from tail biopsies using sodium hydroxide extraction [40], and PCR-based strategies were then used to genotype these mice [41, 42]. All mice used in this study were of mixed genetic background (see Supplementary Table 1).

### Tamoxifen administration

Tamoxifen (Sigma T5648) was dissolved at 20 mg/mL in a 9:1 mixture of corn oil and ethanol the same day of administration to mice. This tamoxifen mixture (500 μL) was administered to mice via oral gavage once per day for 2 consecutive days.

### Allele-specific and quantitative reverse transcriptase PCR

To confirm that exon 14 of *Apc* had been deleted in the prostate of mutant Apc^cKO^ mice, DNA was isolated from whole prostate lobes using the Qiagen DNeasy kit (69504), and PCR amplification was done to amplify wild-type, floxed, or deleted alleles using previously described primers [42]. For quantitative RT-PCR, RNA was removed from mouse prostate using Trizol, centrifuged with chloroform to separate out the aqueous RNA phase, and then RNA was ethanol-precipitated and purified using the Qiagen RNeasy kit (74034). TaqMan^®^ primers and probes for *Axin2* and *Smad4* RT-PCR were purchased and used to perform qPCR in triplicate.

### Histology and immunohistochemistry

Hematoxylin (Sigma MHS16) and eosin (Sigma HT110216) staining and immunohistochemistry was performed on tissues fixed in 10% neutral buffered formalin (NBF) for 24–48 hours, embedded in paraffin, and sectioned at 5 μm thickness. Immunohistochemical staining was optimized using the Discovery XT System (Ventana, Tuscon, AZ). The following antibodies were used for immunohistochemistry: β-catenin (polyclonal, Cell Signaling Technology, Danvers, MA), Ki67 (SP6, Spring Biosciences, Pleasanton, CA), cyclin D1 (SP4, Thermo Fisher Scientific, Halethorpe, MD), AR (EPR1535(2), Abcam, Cambridge, MA), cytokeratin 14 (polyclonal, BioLegend, San Diego, CA), cytokeratin 8 (polyclonal, Spring Biosciences, Pleasanton, CA), p63 (D2K8X, Cell Signaling Technology, Danvers, MA), and Smad4 (B-8, Santa Cruz Biotechnology, Dallas, TX).

### Hormone ELISA

Upon dissection of mice, approximately 500 μL of blood was removed via intracardiac puncture, mixed with 5 μL of 0.5 M EDTA, pH 8.0, and then centrifuged at 6,000 × g for 6 minutes. Approximately 200 μL of serum was removed from the top layer and stored at -80°C. This serum was used in the Cayman Chemical testosterone EIA kit or estradiol EIA kit to determine hormone levels. The student's *T*-test was used to assess potential statistical differences between groups.

### Castration and testosterone re-administration

Surgical castration was performed by making a single 1 cm longitudinal incision into the skin and peritoneum anterior to the preputial gland. The epididymal fat pad, vas deferens, and both testes were removed via cauterization. The peritoneal incision was sutured shut, and the skin was closed with wound clips. Wound clips were removed 14 days post-surgery. Post-castration mice showed no signs of infection. Testosterone (Sigma T1500) was administered to mice via semipermeable silastic tubes subcutaneously inserted into the dorsal mid-trunk region of the mice. This method has been previously described [43]. These tubes were removed when required for testosterone removal.

### Nuance microscopy quantification

Images were taken on a Nuance microscope, and were un-mixed for brown stain and blue (hematoxylin) stain. Identical thresholds were used to quantify the number of positively stained pixels for either the IHC stain (e.g. Ki67) or hematoxylin. Hematoxylin-positive nuclei represented the denominator, and IHC-stained nuclei represented the numerator. The student's *T*-test was used to assess potential statistical differences between groups.

## SUPPLEMENTARY MATERIALS FIGURES AND TABLES


