# Supplementary material for: Deletion of tumor suppressors adenomatous polyposis coli and Smad4 in murine luminal epithelial cells causes invasive prostate cancer and loss of androgen receptor expression
Source: Oncotarget. 2017 May 17;8(46):80265–77. doi: 10.18632/oncotarget.17919 (PMC5655195; doi:10.18632/oncotarget.17919)
Supplement: Supplementary file 1 [file oncotarget-08-80265-s001.pdf]

Deletion of tumor suppressors adenomatous polyposis coli and Smad4 in murine luminal epithelial cells causes invasive prostate cancer and loss of androgen receptor expression

Supplementary Materials

Supplementary Table 1: Genetic backgrounds of mice used in this study

Mouse line	Background
Nkx3.1-creERT	Mixed: C57BL/6 and 129/SV
Apc-flox	Mixed: C57BL/6J and 129SV/J
Smad4-flox	FVB/n
mT/mG	C57BL/6J

Supplementary Table 2: Number of mice used in this study

Experimental group	$Apc^{cKO}Smad4^{cKO}$	$Apc^{flox}Smad4^{flox}$
Tamoxifen-only (Figure 1)	18	17
Tamoxifen+castration (Figure 5)	5	4
Tam+castration+testosterone (Figure 6A)	16	5
Hormone cycling (Figure 6B)	6	7

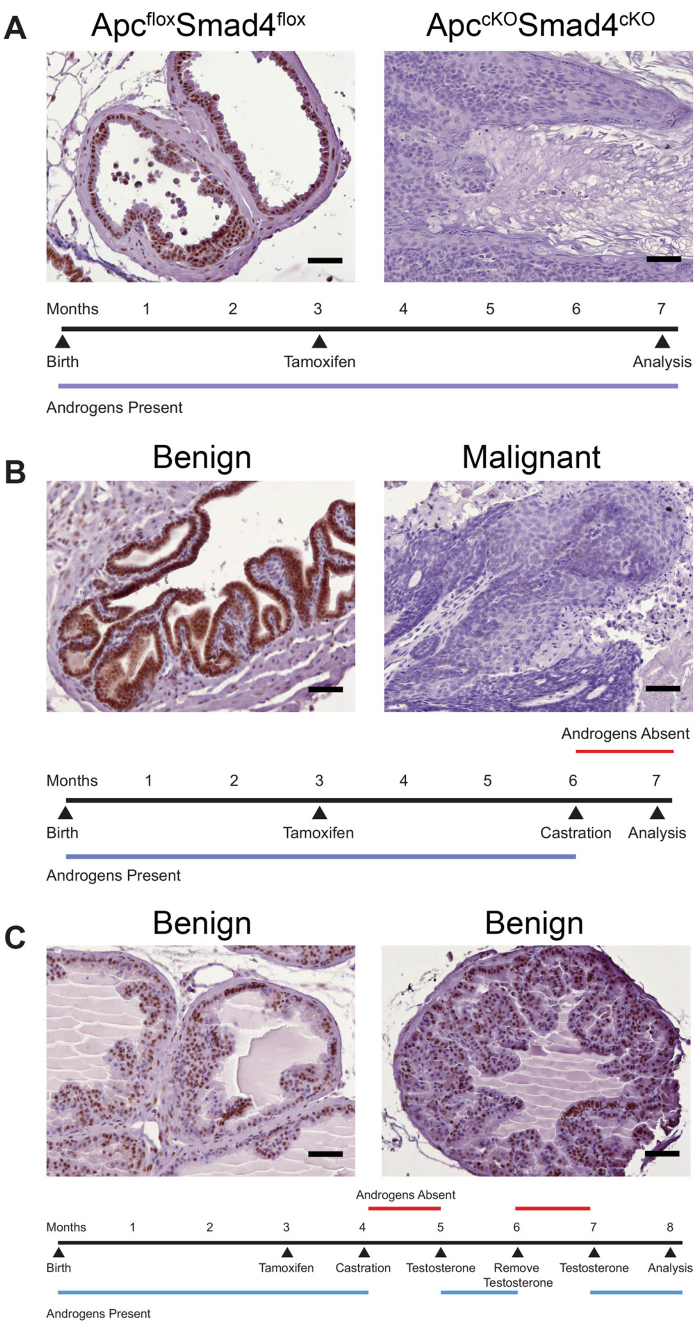

Supplemental Figure 1: AR staining is lost in malignant regions of castration resistant tumors but regained after androgen cycling. Androgen receptor (AR) immuno-staining of prostate tissue from $Apc^{flox}Smad4^{flox}$ and $Apc^{cKO}Smad4^{cKO}$ mice as a positive and negative control of AR staining, respectively, under normal hormone conditions following tamoxifen administration (**A**), benign and malignant regions after tamoxifen treatment and castration (these tumors grow after castration, making them castration resistant) (**B**), and two examples of benign regions after two rounds of androgen cycling (**C**). Scale bars = 100 µm.

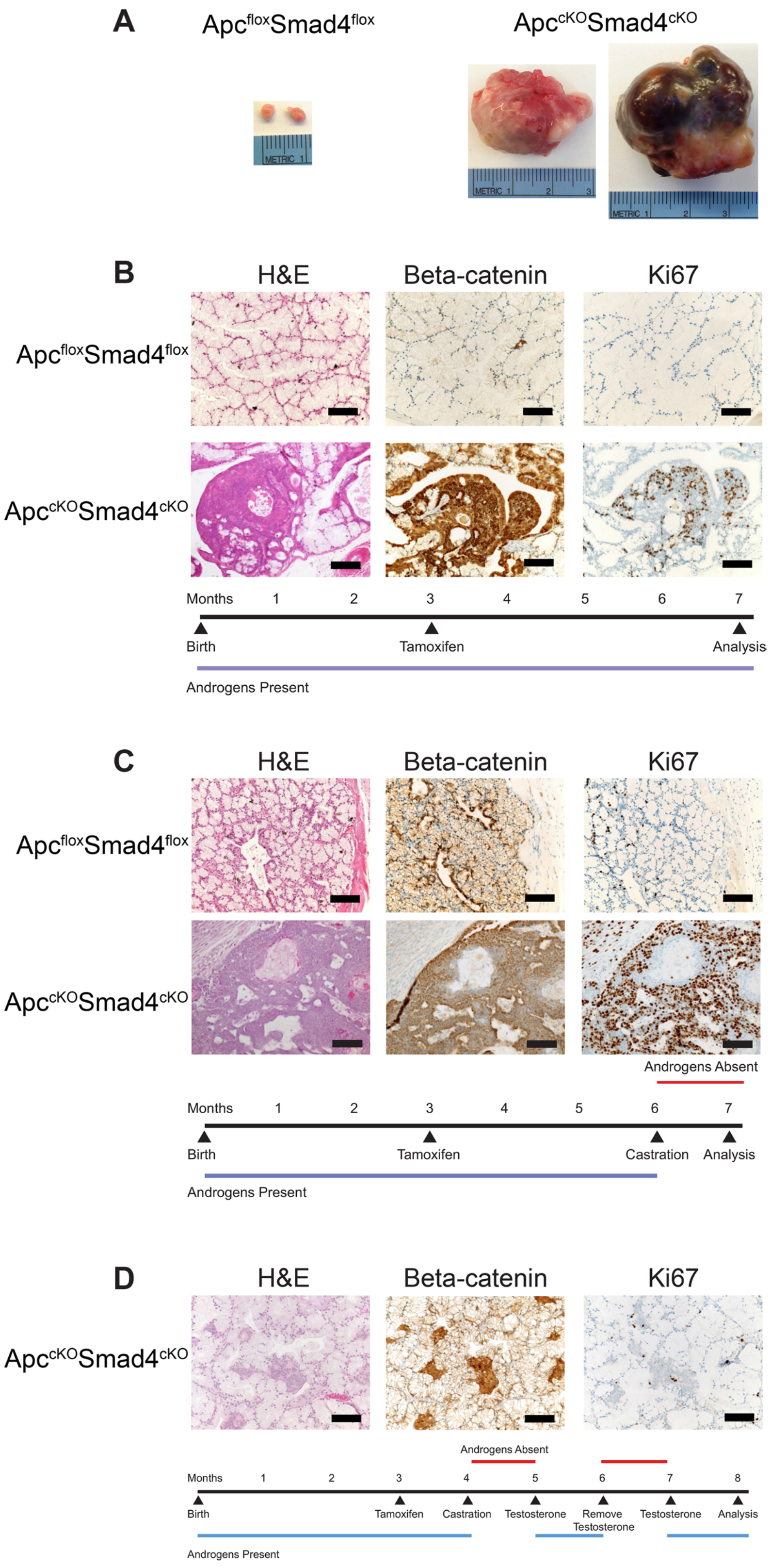

Supplemental Figure 2: Castration resistant carcinoma in bulbourethral gland of Apc^{cKO}Smad4^{cKO} mice, which is reduced after androgen cycling. (**A**) Representative bulbourethral gland (BUG) tumors in Apc^{cKO}Smad4^{cKO} mice compared to normal BUG tissue from Apc^{flox}Smad4^{flox} mice. Hematoxylin and eosin (H&E), beta-catenin, and Ki67 staining of BUG tissue from Apc^{flox}Smad4^{flox} and Apc^{cKO}Smad4^{cKO} mice under hormonally normal conditions following tamoxifen administration (**B**), castration conditions (**C**), and androgen cycling (**D**). Scale bars = 100 µm.

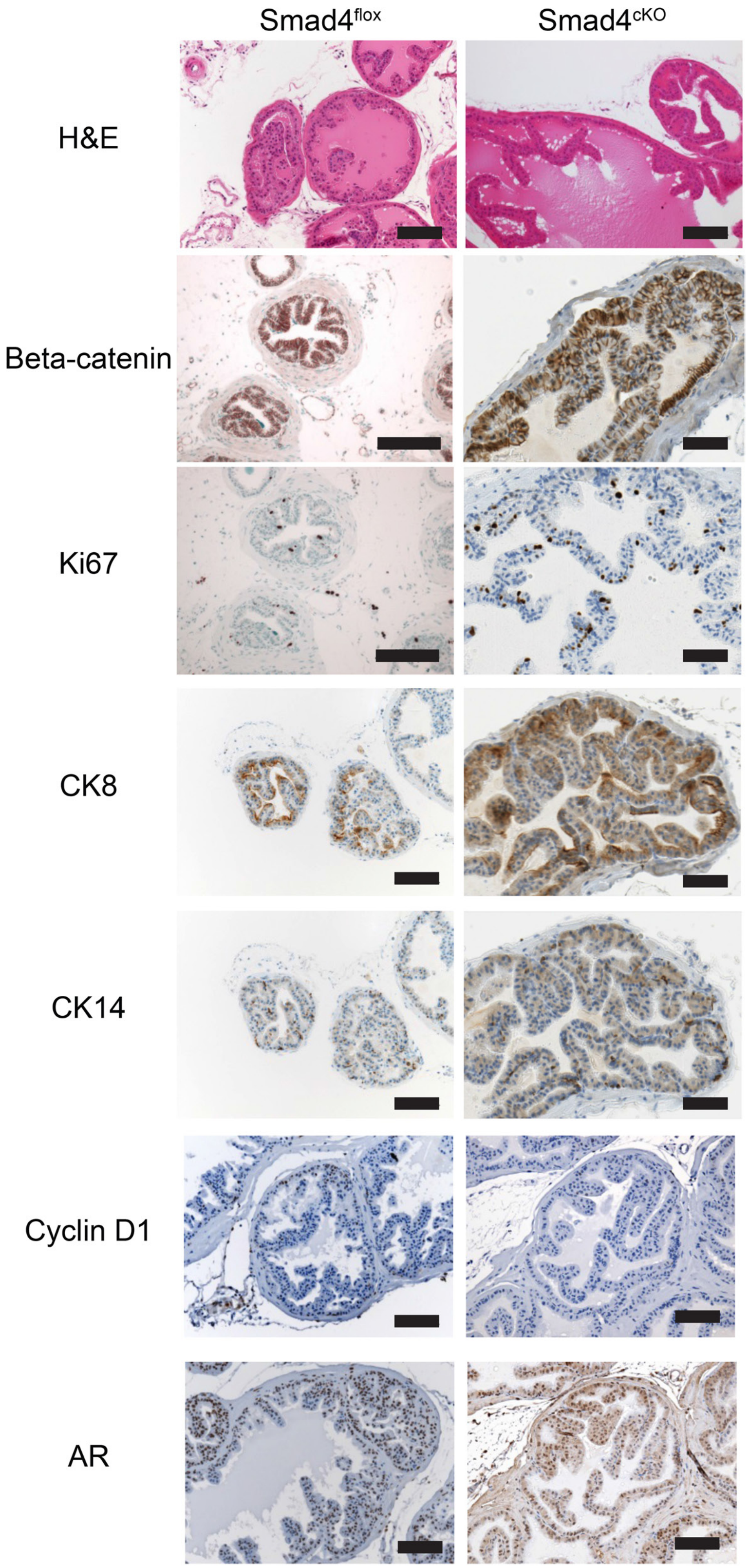

Supplemental Figure 3: No observable phenotype in prostate tissue from Smad4^{cKO} mice. Prostate tissue from Smad4^{flox} and Smad4^{cKO} mice were stained for hematoxylin and eosin (H&E), beta-catenin, Ki67, cytokeratin 8 (CK8), cytokeratin 14 (CK14), cyclin D1, and androgen receptor (AR). Scale bars = 100 μm.
